# Bridging Conflicting Views on Eye Position Signals: A Neurocomputational Approach to Perisaccadic Perception: Eye Position Information in Brain and Model

**DOI:** 10.1111/ejn.70207

**Published:** 2025-08-04

**Authors:** Nikolai Stocks, Fred H. Hamker

**Affiliations:** ^1^ Chemnitz University of Technology Department of Computer Science Chemnitz Germany

**Keywords:** computational neuroscience, corollary discharge, eye positions signals, gain‐fields, perisaccadic perception, saccades

## Abstract

Saccades are an integral component of visual perception, yet the accuracy and role of eye position signals in the brain remain unclear. The classical model of perisaccadic perception posits that the dorsal visual system combines an imperfect eye position signal with visual input, leading to systematic perisaccadic mislocalizations under specific experimental conditions. However, neurophysiological studies of eye position information have produced seemingly conflicting results. One team of researchers observed the eye position signal directly in gain‐field neurons in the lateral intraparietal area (LIP) and found them incompatible with the classical model. In contrast, another team reported evidence for an eye position signal consistent with the classical model, even showing that accurate eye position can be decoded from neural activity. We modeled two subpopulations of neurons in LIP receiving input from two different sources, one representing the corollary discharge containing predictive presaccadic signals, the other representing a slowly updating proprioceptive eye position signal. By decoding eye position from the neural activity of these subpopulations, we observed the model containing sufficient information to allow the decoder to accurately predict and track the perisaccadic eye position. Our findings reconcile the apparent contradiction between the different neurophysiological studies by providing a unified framework for understanding eye position signals in perisaccadic perception. Our results suggest that a combination of a late‐updating proprioceptive signal and a predictive corollary discharge is sufficient for accurately decoding eye position.

## Graphical abstract

In our neuro‐computational study, we modeled LIP gain fields driven by corollary discharge and delayed proprioceptive signals, reconciling conflicting neurophysiological findings. Decoding their combined activity yields accurate eye position information during saccades. Our model advances understanding of perisaccadic eye position signals and their role in visual perception.

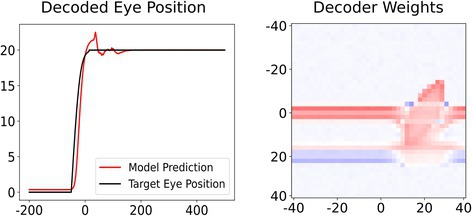

**Abbreviations**
CD: collorary discharge (signal)
EP: eye position (signal)
LIP: lateral intraparietal area
PC: proprioceptive (signal)

## 1 Introduction

**FIGURE 1:**
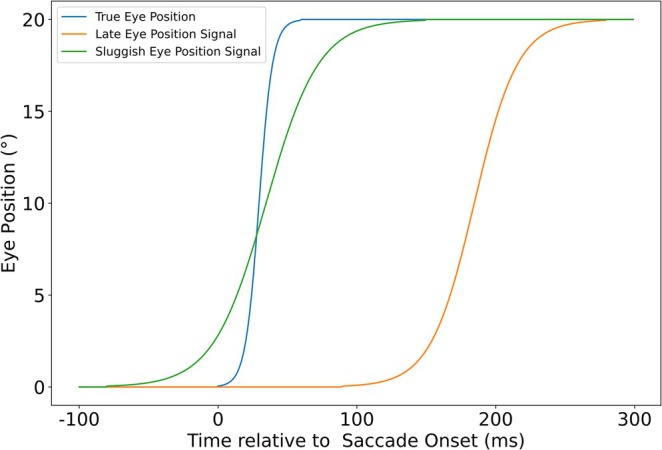
Simplified examples of the sluggish and late eye position signals relative to the true eye position for a 20° saccade. A sluggish, or flat eye position signal precedes the saccade, but begins to fall behind the true eye position during saccade. An eye position signal of this type was observed by Morris et al. (2012). The observations of B. Y. Xu et al. (2012) are sketched by a delayed eye position signal. They observed no systematic increase in activity preceding the saccade, with the eye position signal only reflecting the true eye position after a significant amount of time has passed.

We repeatedly shift our gaze and fixate only for brief periods in time. However, our subjective experience provides us the percept of a stable visual environment. How the brain constructs this percept of stability across saccades is subject to ongoing debates. Evidence points toward the integration of postsaccadic and presaccadic information as well as the usage of internal eye‐related signals from proprioception and corollary discharge (CD) (Bays and Husain 2007; Klier and Angelaki 2008; Hamker et al. 2011; Ostendorf and Dolan 2015; Binda and Morrone 2018). Classically, it has been assumed that the brain only has access to a sluggish eye position signal that indicates a shift in gaze already prior to saccade onset, but at the same time, fully updates only after the end of the saccade (Schlag and Schlag‐Rey 2002; Dassonville et al. 1992). It has been argued that a briefly flashed stimulus is localized relative to such a sluggish eye position signal, resulting in the stimulus being mislocalized in saccade direction when flashed in total darkness prior to saccade and mislocalized against saccade direction when flashed during saccade (Schlag and Schlag‐Rey 2002; Dassonville et al. 1992). Alternatively, an internal eye position may shift the spatial center of retinotopic receptive fields (RFs) (Binda et al. 2009). This eye position signal is often described as a singular signal but may also be a composite effect of multiple signals. The specific role of the eye position signal varies across models, but almost every model contains a set of predictions or assumptions about the nature of the eye position signal which are integral to its ability to explain human perisaccadic perception (for review, see Hamker et al. (2011)). Typically, models rely on a sluggish eye position signal (see green curve in Figure 1) to replicate human perisaccadic perception (Binda et al. 2009; Xing and Andersen 2000). Pola (2004) argued that the assumption of an anticipatory, slow eye position signal could be dropped and replaced by a fast, non‐sluggish signal that starts to change after saccade onset, if visual latency and stimulus persistence are taken into account. However, a late updating postsaccadic signal as observed in gain‐field neurons by B. Y. Xu et al. (2012) (illustrated by the orange curve in Figure 1) would be much later than expected based on the models he discussed.

Studies in monkeys revealed further insights into the disposition of eye position information in visual brain areas. Activity in the parietal cortex and other oculomotor brain areas indicates a preparatory CD triggered by the intended saccade displacement (Colby and Duhamel 1996; Melcher and Colby 2008; Sommer and Wurtz 2008b). Gain fields in the parietal cortex have been shown to contain a proprioceptive tonic eye position signal that encodes the presaccadic eye position during the saccade and only updates after the saccade to encode the new eye position (Wang et al. 2007; Y. Xu et al. 2011; B. Y. Xu et al. 2012). B. Y. Xu et al. (2012) searched for individual gain‐field neurons in the lateral intraparietal cortex (LIP), whose activity directly correlated with the eye position. The neurons that matched their search criteria were classified as consistent; all other neurons were classified as inconsistent. They observed no individual neurons whose activity consistently correlated with the future eye position and only observed a late update of LIP gain fields in consistent neurons.

Morris et al. (2012) recorded extracellular spiking activity from single neurons in multiple visual cortical areas (LIP, VIP, and MT/MST) of macaque monkeys performing saccades and fixations in near darkness. The monkeys were instructed to make a rightward or downward saccade from one of five starting positions to a target stimulus. They analyzed population responses by grouping neurons based on their firing rates, whether they increased or decreased across saccades. By subtracting the responses of both groups, they derived an eye position signal that strongly resembled the sluggish eye position signal seen in Figure 1 across all measured populations. Further, unlike B. Y. Xu et al. (2012), their data showed strong evidence for systematic presaccadic changes in the signals, as neurons encoding the postsaccadic eye position already increased in activity before the saccade, and neurons encoding the presaccadic eye position decreased.

Here, we aim to explore this putative contradiction using a neuro‐computational model of spatial updating in LIP. This model uses gain‐fields to update spatial reference frames based on CD and a proprioceptive eye position signal (PC). This combination of different sources of eye‐related signals allowed us to replicate a range of experimental data (Ziesche and Hamker 2011, 2014; Bergelt and Hamker 2016; Ziesche et al. 2017; Bergelt and Hamker 2019). As detailed in materials and methods, we decoded eye position information from the neural activity in this model, similar to Morris et al. (2016) from their neural recordings, and also compared the updating of LIP model gain fields to experimental data from B. Y. Xu et al. (2012). We find that the model is consistent with both observations. The model explains this by its ability to temporally integrate different sources of eye position, such that correct eye position can be decoded by weighted averaging, even when some gain field neurons update late after saccade.

## 2 Materials and Methods

### 2.1 Overview of the Neurocomputational Model

This study builds on a neurocomputational model first published by Ziesche and Hamker (2011). Unlike previous models, which often modeled a somewhat arbitrary singular eye position signal, this model aimed at considering those signals that are known to exist somewhere in the brain. One obvious signal is an efference copy (Von Holst and Mittelstaedt 1950) or, similarly, a CD (Sperry 1950), which is a preparatory signal for an upcoming saccade. Neurons in the superior colliculus (SC) and the frontal eye field (FEF) such as so‐called movement or burst cells, can be classified as representing a CD (Hamker et al. 2011). They code for eye displacement in a retinocentric reference frame, increase in activity prior to the saccade, and peak around saccade onset (Sommer and Wurtz 1998, 2004, 2008a, 2008b). Thus, the CD represented by those neurons has a clear and well‐known spatiotemporal profile. A second obvious signal is proprioceptive and has been observed in the somatosensory cortex (Wang et al. 2007; Y. Xu et al. 2011). It updates to the new eye position briefly after saccade offset. However, B. Y. Xu et al. (2012) reported an even further delayed update in LIP gain‐field neurons. This original model by Ziesche and Hamker (2011) accounted for perisaccadic mislocalization of flashed stimuli in total darkness by integrating visual and both eye‐related signals within a spatially organized cortical map. In a subsequent study, Ziesche and Hamker (2014) used the same framework to explain the emergence of predictive remapping in area LIP and its influence on perceived spatial stability as measured by the so‐called saccadic suppression of displacement task. These experiments require subjects to localize a displaced saccade target. Data show that subjects detect the displacement if the displaced target is blanked for 150 ms and reappears after the saccade. Ziesche and Hamker (2014) explained this finding by an inherent peri‐saccadic stabilization within a dynamic neural network. Bergelt and Hamker (2016) investigated predictions of this model with respect to suppression of displacement detection also in the absence of eye movements. Ziesche et al. (2017) investigated in detail how the model can account for different experimental variations in saccadic suppression of displacement tasks. Finally, Bergelt and Hamker (2019) expanded the model to two spatial dimensions and applied it to explore spatial updating of attention across eye movements, showing that a dynamic interplay of CD and visual input could account for observed attentional facilitation at both presaccadic and postsaccadic target locations.

The model investigated here is built and simulated using the neurosimulator ANNarchy 4.7.2 (Vitay et al. 2015). At the model's core are gain‐field neurons, whose responses are determined by a visual signal from early visual processing stages and by various eye‐related signals: the delayed, craniotopic proprioceptive signal (PC), the phasic, retinotopic CD of the planned saccade, and additional feedback projections.

The mechanism of coordinate transformation within the gain‐field populations is inspired by the multiplicative basis function approach (Pouget et al. 2002), which can be interpreted as the net effect of many LIP gain‐field neurons with sigmoid activation functions (Bergelt and Hamker 2019). The firing rate of a LIP gain‐field neuron is dynamically updated and shaped by both forward and feedback projections (see simplified rate equation in Equation (1)), where *x* is the location of a visual stimulus and *y* is an eye position signal. The eye function *g(y)* acts as a multiplicative gain on the RF response *f(x):*

(1) $$r\left(x,y\right)=\left(f\left(x\right)\right)\cdot \left(C+g\left(y\right)\right)\cdot \text{Feedback}+\text{Noise}$$

The constant C ensures that gain‐field neurons still react to stimuli presented in their RF in the absence of a signal *g(y)*, which is particularly relevant for the transient CD signal.

Our proposed model does not require an explicit unitary eye position signal; instead, LIP neurons are influenced by one or both eye‐related signals. For simplicity, we define two separate populations: The LIP_PC_ is modulated by the tonic proprioceptive (PC) signal, while LIP_CD_ is modulated by the transient CD signal. The separation of the LIP into two distinct neural populations is not essential to the model's function (see Supporting Information).

**FIGURE 2:**
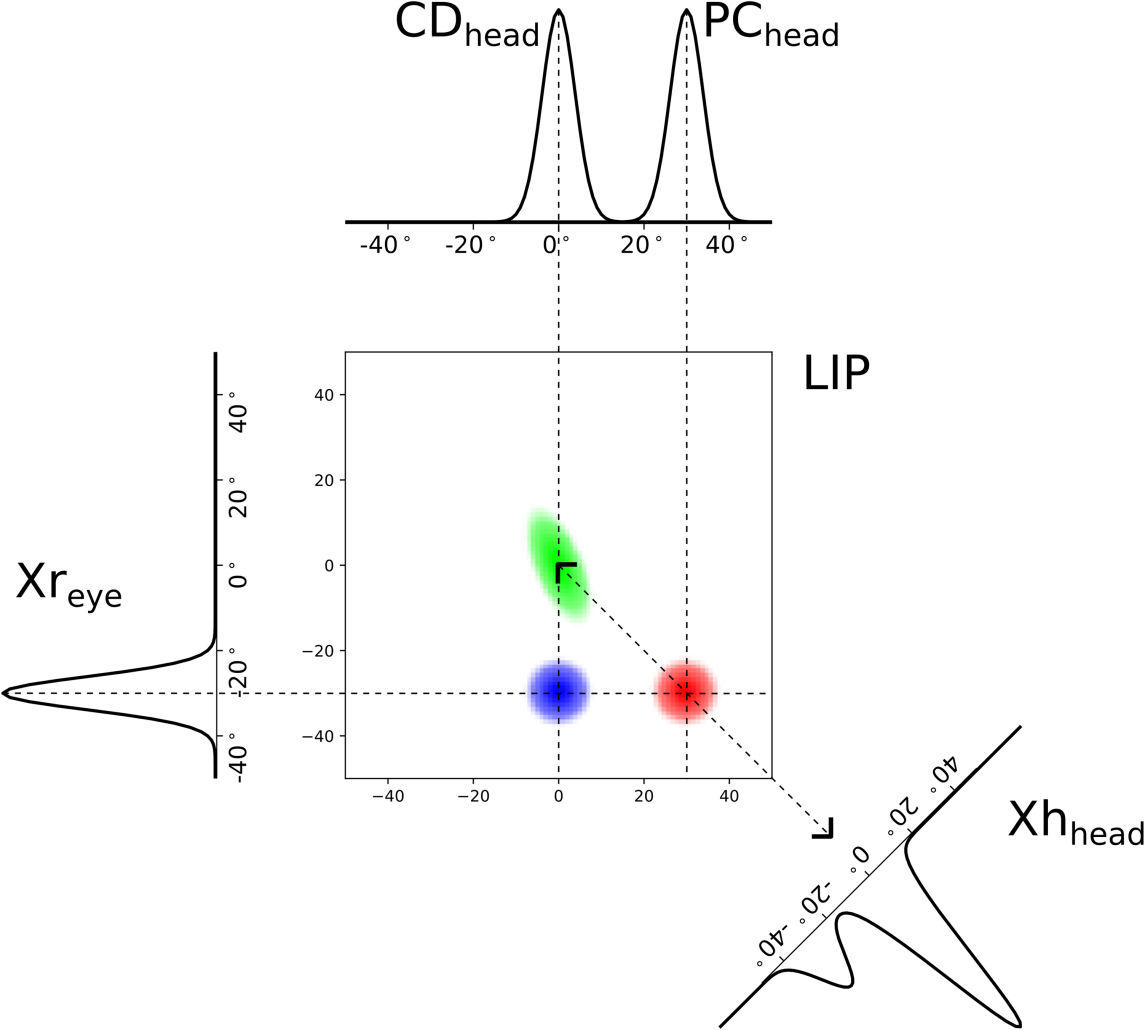
Schematic overview of LIP gain field dynamics to show the multiplicative basis function approach outlined in Formula (1). Signals with different base‐coordinate systems are fed along separate axes into a population with a higher dimension than the input signals. This larger population represents the space of all possible ways to combine the two input signals. This means that any hidden information contained in the interaction of the input signals must show itself as an identifiable pattern of activations in this higher‐dimensional population. For easy visual interpretation our gain field populations and inputs are arranged in such a way, that neurons encoding the same craniotopic stimulus position are found along the main‐diagonals of the gain field population. Further, both eye related inputs are shown on a single axis.

While the original model of coordinate transformations using gain‐fields (Pouget et al. 2002) considered conditions with static eye positions, we expanded this concept toward the full dynamics during eye movements. A simplified sketch (see Figure 2) illustrates the dynamic gain‐field approach with respect to a tonic proprioceptive (PC) signal and a transient CD signal. A map of LIP gain‐field neurons responds to combinations of stimulus and eye position signals resulting in multiple transient activity bumps around the saccade onset. Long before saccade onset, the CD signal is inactive, and the PC signal encodes the present eye position resulting in the red activity bump. A diagonal readout creates a population response in Xh_head_ that encodes stimulus position within a craniotopic reference frame. Prior to the saccade, the CD signal ramps up and peaks around the saccade onset leading to the blue activity bump. The interaction of the head‐centered population Xh_head_ with the CD signal creates a third activity bump (green) that leads to transient effects known as predictive remapping, as it maps the head‐centered response backwards to its anticipated cause in the reference system of the future eye position. The latter population response explains effects such as RFs shifting in the direction of the saccade vector prior to saccade onset (Ziesche and Hamker 2011).

**FIGURE 3:**
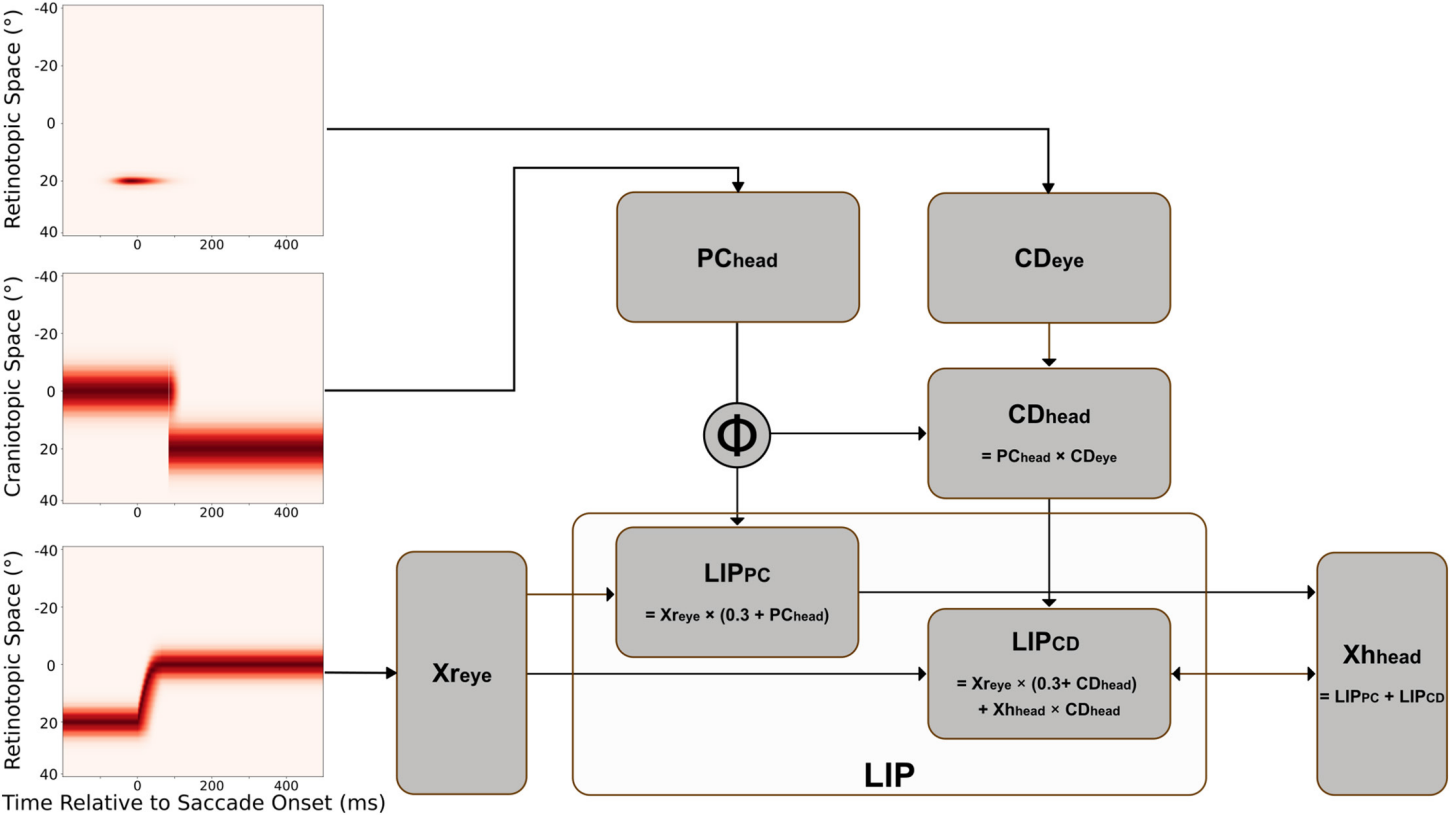
Overview of the model. The model receives three different signals. The corollary discharge (CD) into CD_eye_, which is transient, peaks around saccade onset, and is spatially centered on the saccade target location in retinotopic space (eye displacement) (Sommer and Wurtz 1998, 2004, 2008a, 2008b). It already builds up prior to saccade by 50 ms and decays after saccade onset. The signal driving the PC_head_ population represents proprioceptive (PC) eye position information. It updates after the saccade. The outgoing projections of the PC_head_ are delayed to account for the observation that LIP gain‐field neurons update eye position late after saccade (B. Y. Xu et al. 2012) using a randomly sampled delay from the distribution *ϕ* for each connection. The third input arrives from the visual stimulation, the retinotopic Xr_eye_ response, here from the saccade target stimulus, which is delayed by 30 ms to account for visual latency. The CD_head_ population transforms the CD signal from a retinotopic to a craniotopic signal, so that LIP_CD_ and LIP_PC_ operate in the same coordinate system. LIP_CD_ and LIP_PC_ maps are at the core of the model as they dynamically integrate stimulus with eye information. LIP_CD_ also receives input from the Xh_head_ population, which is crucial for the predictive remapping described in Ziesche and Hamker (2011).

The temporal dynamics of these eye‐related signals are shown in Figure 3. The behavior of LIP_PC_ is inspired by data from neural recordings in LIP (B. Y. Xu et al. 2012). Previous versions of the model implemented the proprioceptive PC signal by simply updating the driving input of the PC_head_ population after the saccade, which resulted in a sudden and complete stepwise shift of the PC signal. In our present version, all outgoing projections are individually delayed between 120 ms and 250 ms using random delays drawn from a normal distribution *ϕ* (*μ* = 200; *σ* = 100; min. delay = 120 ms; max. delay = 250 ms) to mimic more variability with respect to the eye position update. We chose this implementation for a few reasons. First of all, the previous stepwise update of the PC signal is strongly simplified. While this is in principle not a problem in the previous model, in our present study the decoder could rely on this deterministic signal and would become too powerful. By randomizing the delays for each PC signal at the synaptic level in each run, we force the decoder to produce a coherent response to all signals in the neural population over its entire duration. The modeled visual space is simplified to a single spatial dimension and ranges from −40° to +40°, covered by 40 neurons whose RFs overlap, with RF centers separated by 2°. The two‐dimensional gain‐field maps contain 40 × 40 neurons.

### 2.2 Simulations and Data Analysis

We collected data from our model in an experimental setup similar to the one used by Morris et al. (2012). In their version, monkeys were trained to perform a saccade to the target stimulus as soon as it was presented. As a result, the visual transient and saccade onset are potentially strongly correlated in their dataset. To account for observed variabilities in saccade latencies and prevent the strong correlation of stimulus onset and saccade onset, we model a variable delay between the appearance of the target stimulus and saccade execution. The models' saccades are generated using a modified version of the mathematical description of saccade trajectories found in Van Wetter and Van Opstal (2008), which captures the nonlinear relationship between saccade amplitude and velocity. We used saccades of 20° amplitude to the right with a uniform saccade duration of 62 ms. In addition to the stimulus drive, we add low, additive stochastic noise to both LIP populations that mimics the natural fluctuation of neural baseline firing activity.

This experimental design also allowed us to perform a comparison of model simulation data with experimental data from B. Y. Xu et al. (2012). We analyzed our data by following their procedure, calculating a gain field index (GFI) to express the influence of the PC signal across saccades:

(2) $$\mathrm{GFI}\left(t\right)=\left(V_{\text{probe}}\left(t\right)-V_{\text{poststeady}}\right)/\left(V_{\text{presteady}}-V_{\text{poststeady}}\right)$$

B. Y. Xu et al. (2012) separately calculated the GFI for neurons that either experienced a gain increase or decrease. *V*_presteady_ and *V*_poststeady_ represent the steady response of the gain field either before or after the PC update, with *V*_probe_ being the current response. For the GFI to produce meaningful values, either the experimental design or the neuron selection needs to ensure that the difference between *V*_presteady_ and *V*_poststeady_ is entirely due to changes in the PC signal. The GFI takes a value of zero if the neuron's response reflects the postsaccadic eye position, and a value of one for the presaccadic eye position, while partially completed PC updates result in values between 1 and 0.

### 2.3 Decoder

**FIGURE 4:**
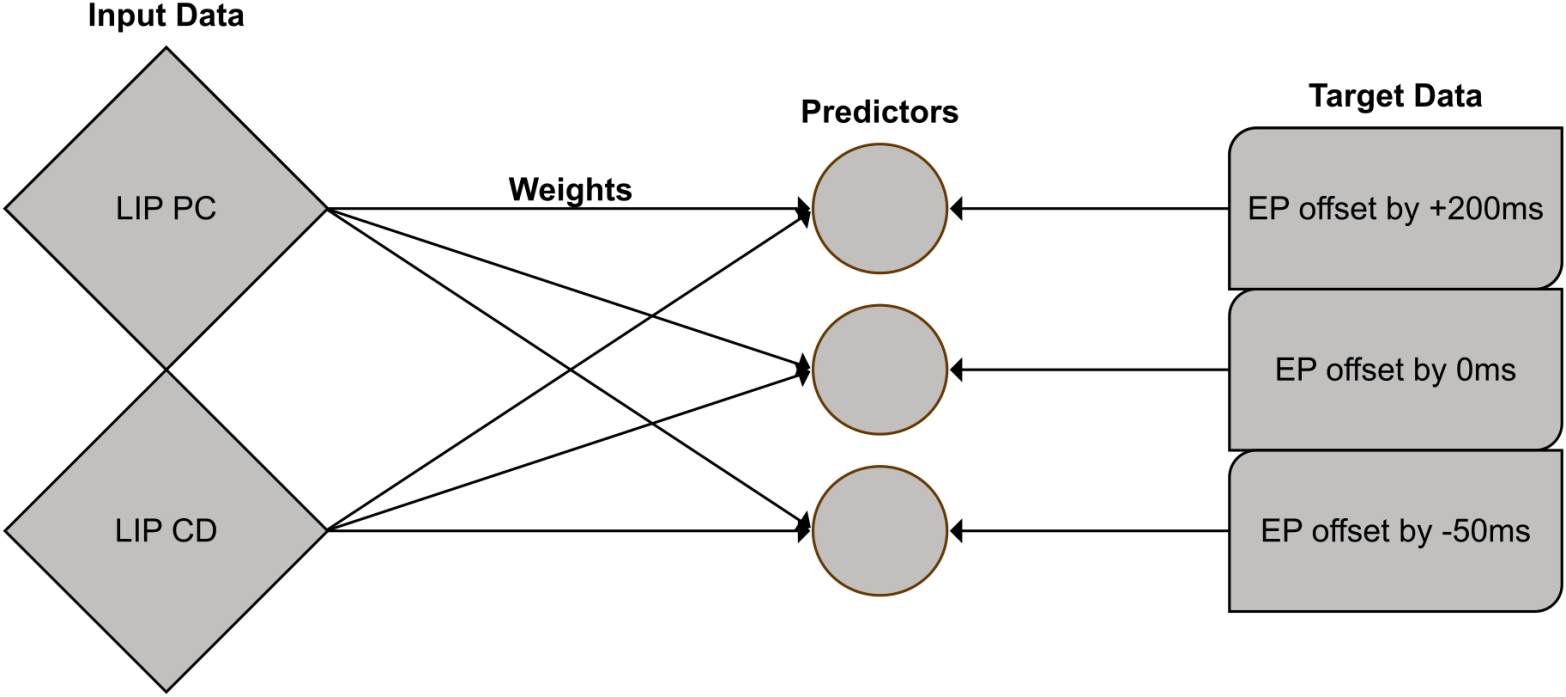
Overview of the single dense layer decoder for the eye position (EP) trajectory and its temporally offset copies. The input is the firing activity of all neurons in LIP. The weighted sum calculated in each integrator directly represents the particular eye position. The input weights are trained based on the mean square error between the predicted EP and the target EP.

To extract eye position information from our model's neural activity, we applied the same general procedure as Morris et al. (2016) and constructed the linear decoder seen in Figure 4, using Keras (Version 2.12.0) and a rectified linear activation function (ReLU). In our emulation of the experiment, the model executes saccades, which we record along with the network's activity. Since the saccade itself can be approximated as a series of eye positions, the recordings consist of paired sequences of discrete network states and eye positions at each timestep of the numerical solution to the model's ordinary differential equations. Like Morris et al. (2016), we use the network recordings as input and the eye position records as target. The decoder's task is to find a set of static weights that result in the weighted sum of the input equaling the target eye position. By succeeding, it proves that the eye position information is not only contained in the network activity but also that it is accessible by a linear readout. Like Morris et al. (2016), we can expand this process to test for the ability to predict and remember the eye position. This is done by adding a positive or negative temporal offset to the target eye position while leaving the model input unchanged. The linear decoder serves as a theoretical probe. Its role is to define a lower bound on decodability. If eye position information cannot be recovered by a linear classifier, it is unlikely that any biologically plausible mechanism could reliably extract it under those conditions.

## 3 Results

**FIGURE 5:**
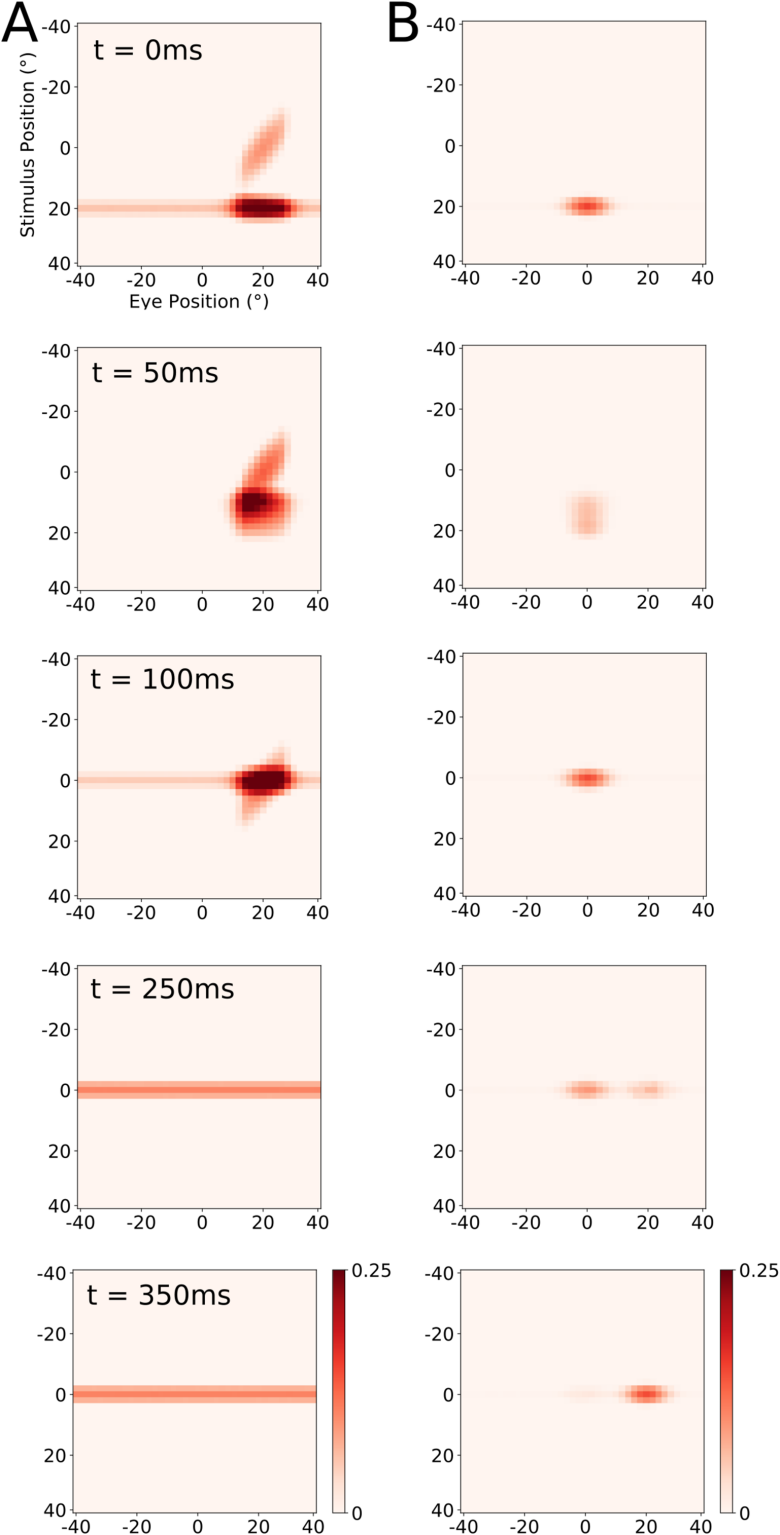
Neural activity in both LIP gain field maps, with time aligned to saccade onset. Neurons are sorted according to their selectivity for eye position (*x* axis) and their selectivity for stimulus position (*y* axis). (A) LIP_CD_ Map. The horizontal line of activity is caused by a response to the visual stimulus. Further activity is induced by the stimulus and eye position gain and its remapped activity prior to saccade onset. (B) LIP_PC_ Map. The LIP_PC_ population is driven by the visual stimulus that is gain modulated by the PC signal that updates late after saccade.

Figure 5 shows the typical neural activity in the two LIP gain fields during our simulation of the simple saccade task studied in monkeys by Morris et al. (2012). Figure 5A shows activity representing a combination of the CD induced future eye position at the saccade target that increases stimulus gain. Predictive remapping emerges via the pathway of Xh_head_ and induces prior to saccade an additional blob of increased neural activity. Further detail about how the model accounts for predictive remapping of RFs, see Ziesche and Hamker (2011, 2014), and predictive remapping of attention, see Bergelt and Hamker (2019). During and after saccade, the CD signal declines, leaving only the baseline activation of the saccade target stimulus. The PC signal is initially centered on 0° and updates after the saccade to 20° (Figure 5B). The LIP_CD_ map is highly dynamic and at t = 0 already contains the remapped stimulus in the future eye reference frame. Only once the pre‐ and intrasaccadic periods have passed, and the CD signal decayed, does the LIP_PC_ map contribute accurate information that is not anymore present in LIP_CD_. With this in mind, one would expect a decoder trained on this dataset to largely ignore the LIP_PC_ map for all but the postsaccadic period.

**FIGURE 6:**
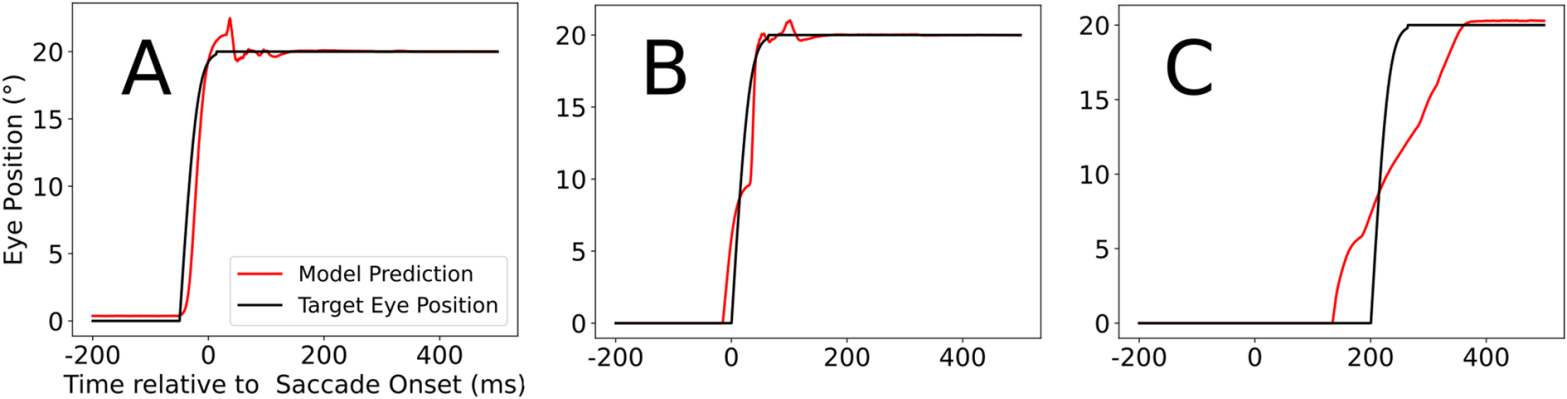
Response of the decoders targeting the future (A), current (B), and past (C) eye position. The black graph represents the target EP, while the red graph represents the decoder's output.

Figure 6 shows the decoder's output (red) and the target eye position (black). The decoder is largely accurate for the future (Figure 6A), as well as for the current eye position (Figure 6B), while failing to extract an accurate past eye position (Figure 6C). This indicates that the CD signal plays the pivotal role in enabling the decoder to extract eye position information, which aligns with our expectations.

**FIGURE 7:**
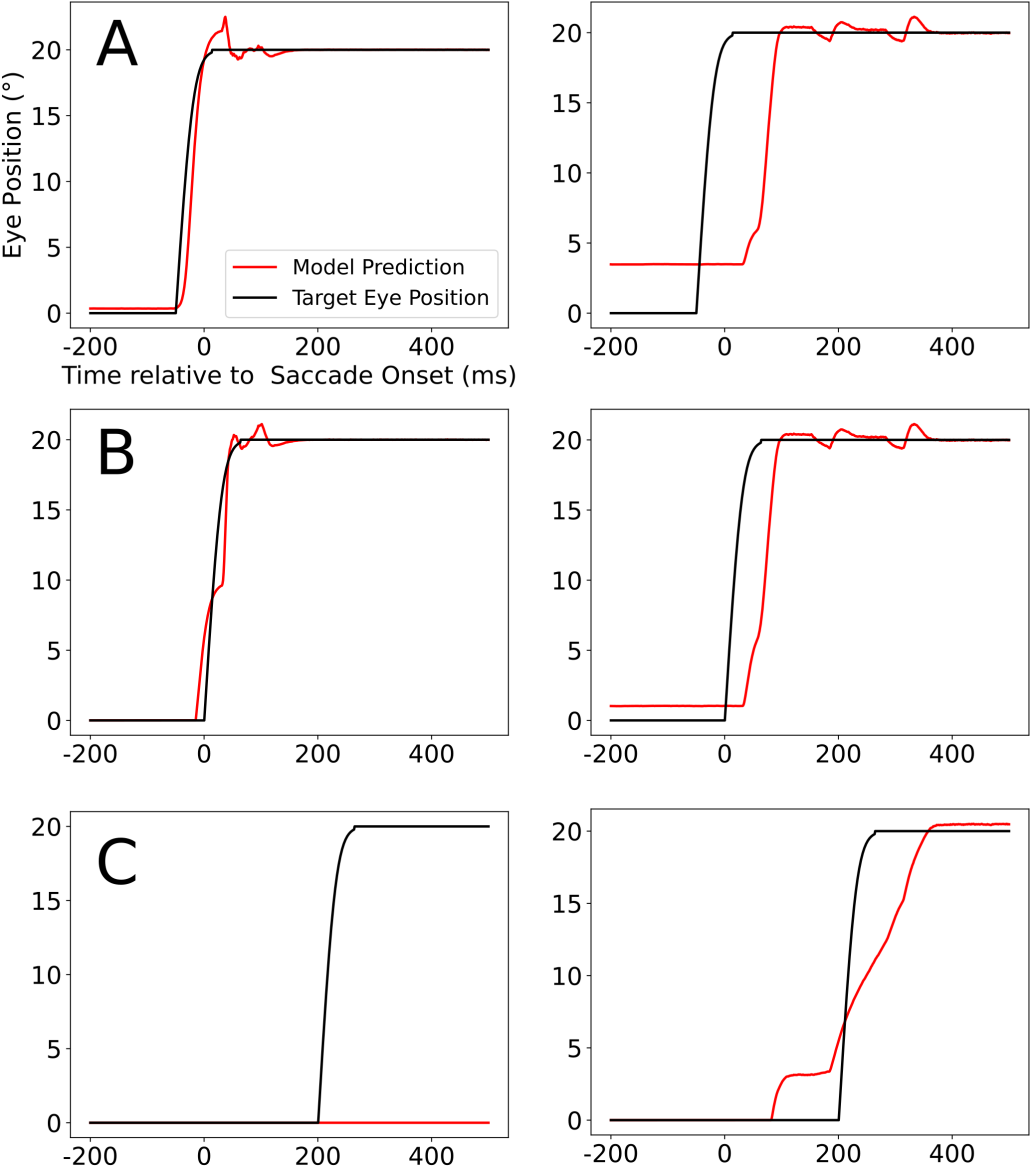
Response of the decoder using only one of the two LIP maps as input data (LIP_CD_ on the left, LIP_PC_ on the right) targeting the future (A), current (B), and past (C) eye positions. The black graph represents the target EP, which the decoder is trying to match, while the red graph represents the decoder's output.

When we train the decoder to perform the same task, but now only using either the LIP_CD_ or LIP_PC_ activity, the decoder's performance for the future and current EP is essentially not influenced by the absence of the LIP_PC_ population, while LIP_CD_ seems to contain practically no information related to the past eye position (Figure 7).

**FIGURE 8:**
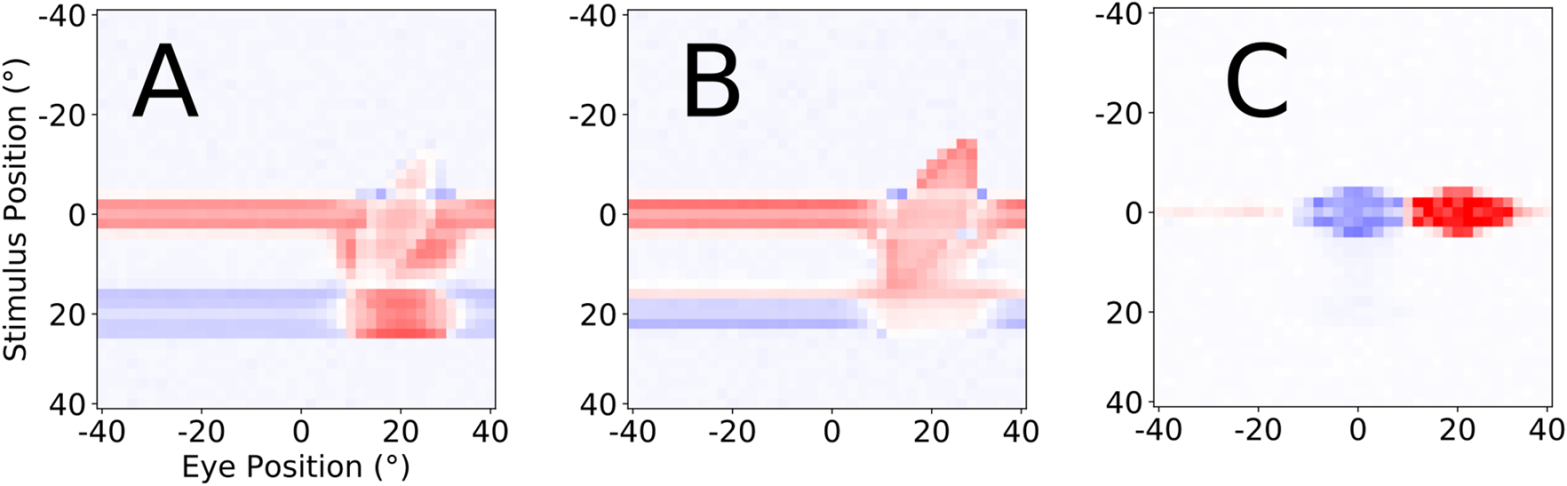
Relevant, informative weight maps that contribute to the decoding of the future (A), present (B), and past (C) eye position. In (A) and (B), we only show LIP_CD_, and (C) shows LIP_PC_. Red color signifies positive and blue color signifies negative weights.

By analyzing the structure of the learned weight‐matrices, we can understand how the decoder produces its outputs, and when it fails to deliver an accurate eye position signal (Figure 8). Due to the variability in saccadic latency, both decoders need to initially identify the saccade onset without the aid of visual onset information. The future eye position decoder assigned strong positive weights to the activity directly caused by the CD signal. The current eye position decoder reacts strongly to the CD‐driven predictive remapping (feedback via Xh_head_).

Weight gradients are mostly found along the vertical, that is, the retinotopic visual axis. That means that the decoder, while the CD signal is active, interprets visual change as directly caused by the saccade. In other words, for the duration of the CD signal, visual stimuli are treated as stationary relative to the body, which means that the change in eye position is directly encoded in the retinotopic stimulus‐driven activation. The reason for the inaccurate (sluggish) past eye position estimate (Figure 6C) becomes apparent upon analysis of Figure 8C. As the PC update, which drives the postsaccadic change in activity in LIP_PC_, is randomized for each neuron and each trial in the LIP_PC_ population, this cross‐trial randomization decorrelates the individual neurons activity from the saccade onset within the bounds set by the distribution of the PC update. Put differently, viewed over all trials, each individual neuron activity increase only reliably encodes that the saccade happened at least 120 ms ago, which is the minimum value of the PC update. This avoids having specific neurons reliably encode specific offsets. To the best of our knowledge, no experimental study has systematically analyzed the single neurons' prediction of saccade onset. If saccade onset were fully predictable on the basis of a single neuron activity, the eye position prediction would become trivial, simply requiring the decoder to identify the neuron that most closely aligns with the target data. Yet, despite this randomization, the information needed to reconstruct the target eye position 200 ms ago is still contained in the overall population response and would thus be accessible to a higher‐order decoder, yet is beyond the limits of what can be expressed through the application of static weights to individual neurons by a linear decoder. The decoder still attempts to reduce the mean square error as much as possible, which is why we see two homogeneous clusters of weights, one at the presaccadic eye position (negative), and one at the postsaccadic eye position (positive) in Figure 8C, as this is simply the representation of an unstructured response to a signal with no decipherable internal structure. The fact that the equally weak linear decoder of Morris et al. (2016) succeeded where ours failed hints at a difference in signal structure. Specifically, this implies that certain neurons or groups of neurons in the recordings used by Morris et al. (2012) reliably encode the new eye position with a stable offset relative to the saccade.

**FIGURE 9:**
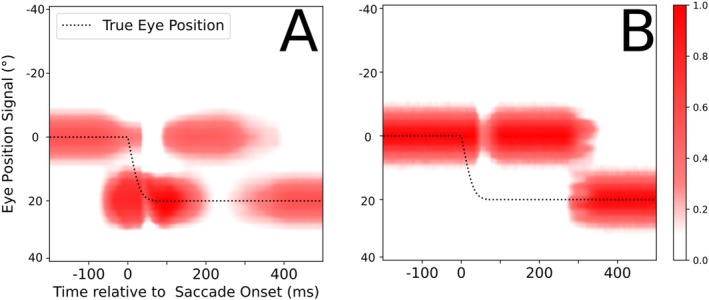
Visualization of eye position information in the LIP gain fields. To create this visualization both LIP maps have been normalized, then added together. Only the slice of neurons showing the highest activity is shown for each timestep. (A) Eye position encoding gain field activity using our models default setup. The CD signal and the accelerated gradual onset of the PC signal provide a robust foundation for a fluent transition of eye position information across the saccade. (B) Eye position encoding gain field activity using parameters modeled after the observations of B. Y. Xu et al. (2012). No predictive activity either prior or during the saccade, and a late and sudden update of the eye position signal, resulting in a perisaccadic mismatch between perceived eye position and post‐saccadic stimulus position. Without visual references, the resulting sensory input is indistinguishable from movement of the retinal stimuli in the craniotopic frame of reference.

Predictive remapping maps stimulus‐induced neural activity into the future reference frame and thus anticipates the consequences of the saccade. It arises in our model from the feedback signal of the pre‐saccadic craniotopic stimulus position encoded by Xh_head_, and the CD signal arriving via CD_head_ (see the green population activity in Figure 2). Thus, predictive remapping in the model could improve eye position decoding as its neural correlate provides additional information for the decoder. Figure 9 illustrates the additional eye position information introduced through predictive remapping before it is processed by the decoder. To further test this, we repeat the experiment and training of the decoder, but now with a model where the feedback projection from Xh_head_ back to LIP_CD_ has been removed.

**FIGURE 10:**
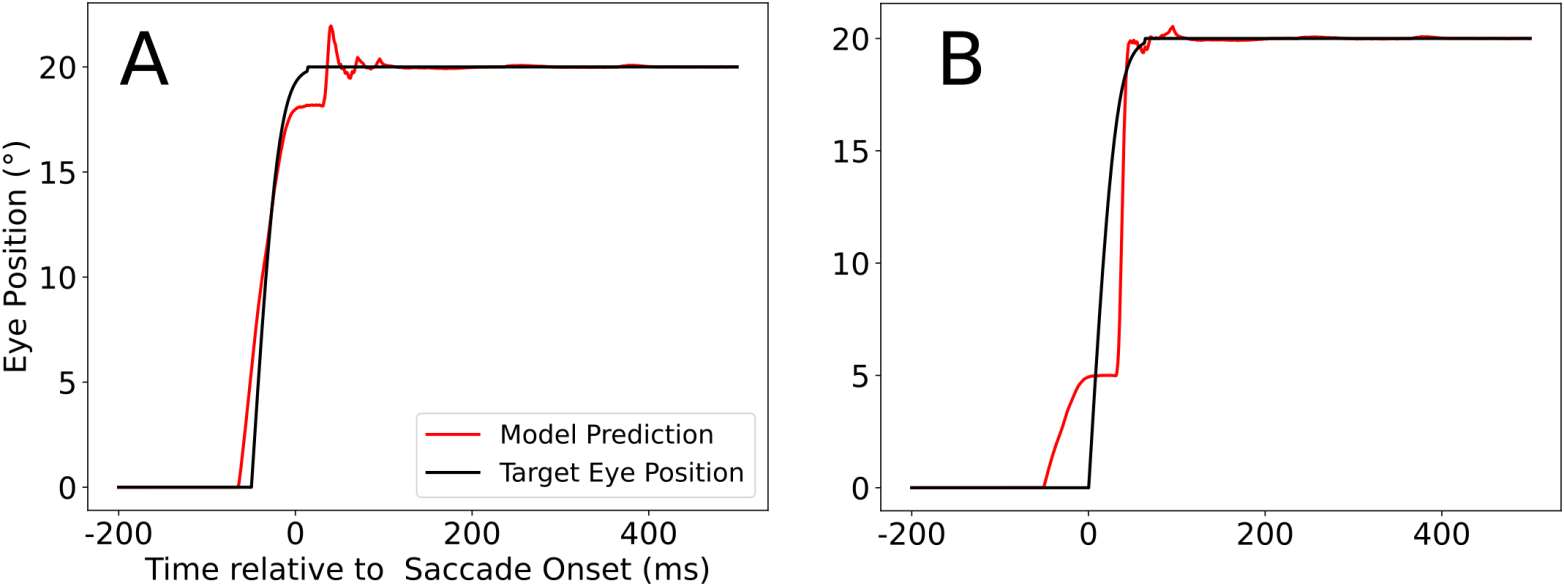
Response of the decoder when trained and tested on a version of the network without predictive remapping. Decoding is shown for the future (A) and the current (B) eye position.

**FIGURE 11:**
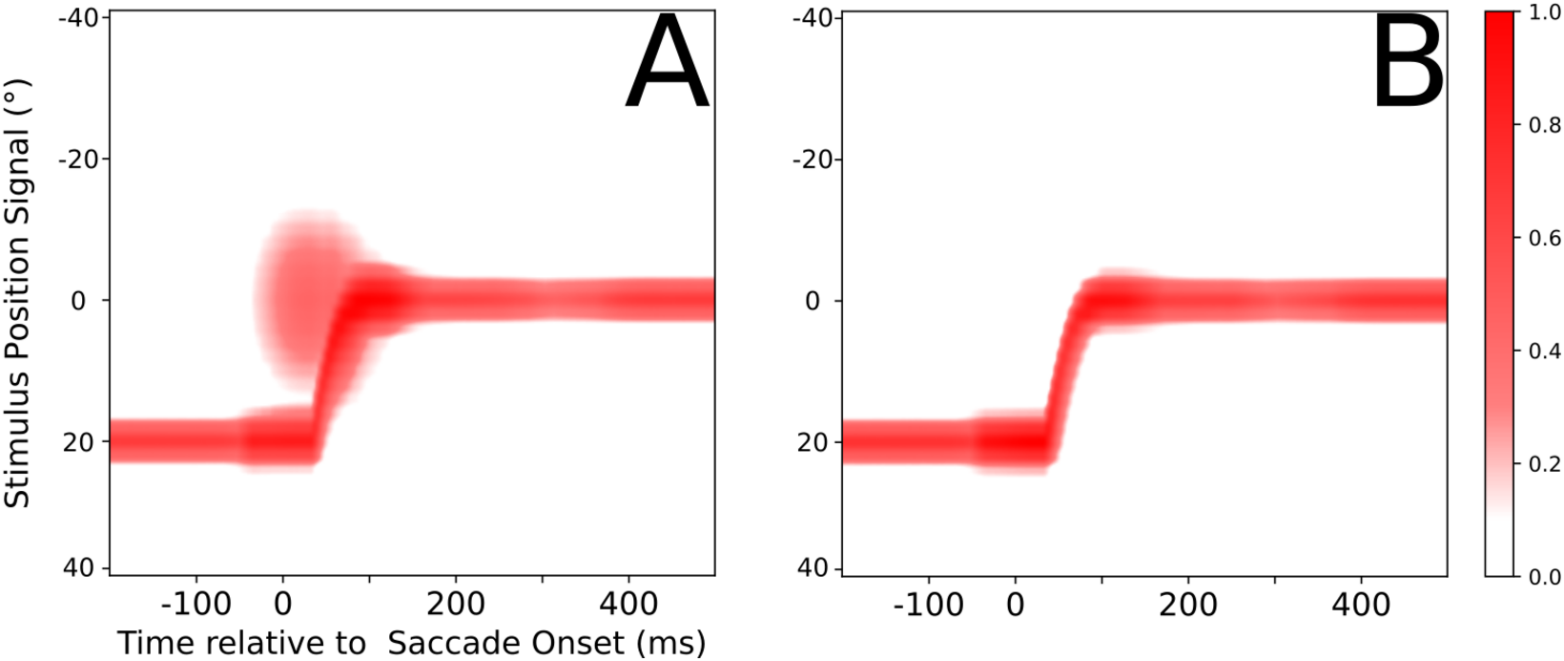
Visualization of retinotopic stimulus position information in the LIP gain fields. We use the same process used to create Figure 9 to show the retinotopic Stimulus encoding activity in both LIP Maps over time with (A), and without (B) predictive remapping.

In Figure 10, we observe that the ability to predict the future eye position is not meaningfully altered by the removal of predictive remapping. This is mostly due to the CD signal onset being similarly timed with the temporally offset saccade onset. However, predictive remapping improves decoding the current eye position, specifically to detect the saccade onset. To illustrate the effect of predictive remapping, we compare the model with predictive remapping to the one without predictive remapping with respect to its neural activity encoding stimulus position (Figure 11). The model with predictive remapping anticipates future stimulus position already prior to saccade onset.

**FIGURE 12:**
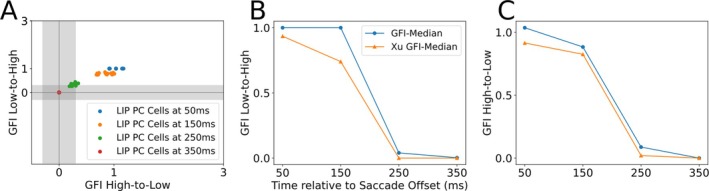
Network GFI Scores of LIP_PC_ neurons. (A) Temporal change of average GFI for LIP_PC_ neurons. (B) Median GFI for low‐to‐high neurons. Model GFI scores are in blue, and experimental data are in orange. (C) Median GFI scores for high‐to‐low.

Finally, we verify if the LIP_PC_ population is consistent with the data provided by B. Y. Xu et al. (2012). We calculate the GFI scores as defined in the Methods section. The neurons chosen for this calculation need to fulfill the following criteria, similar to the ones of B. Y. Xu et al. (2012). They are required to change their postsaccadic activity purely because of changes in the PC signal. The subset of neurons that fulfill the criteria is visible in Figure 5B, at *t* = 100 ms and *t* = 350 ms, respectively, as they experience a gain increase or decrease due to the PC signal update. The neurons that experience a gain decrease due to the PC update, seen active in 5B at *t* = 100 ms, are used to calculate the high‐to‐low GFI values, and the ones experiencing an increase, seen active in 5B at *t* = 350 ms, are used to calculate the low‐to‐high values. The comparison between the GFI scores in Figure 12 shows that the late updating behavior of our LIP_PC_ gain fields closely matches observations by B. Y. Xu et al. (2012).

## 4 Discussion

Recent studies analyzed eye position information in area LIP. B. Y. Xu et al. (2012) have not found direct evidence of individual LIP gain‐field cells carrying predictive information. Neurons that showed systematic changes in activity linked to changes in eye position indicate a late update of eye position gain after a saccade, as measured by a gain field index. In contrast, the recordings of Morris et al. (2012) show increases in the activity of neurons encoding the postsaccadic eye position before saccade onset. This presaccadic increase in activity likely originates from a CD, observed in different brain areas (Duhamel et al. 1992; Sommer and Wurtz 2004). In addition, Morris et al. (2016) have demonstrated that accurate predictive information exists in LIP at the population level. However, the experimental design of Morris et al. (2012) potentially introduces a correlation between the visual saccade target stimulus and the saccade, as the monkeys were instructed to perform a saccade immediately after the target appeared and as the stimulus remains visible. It is unclear to what extent the decoder's strong performance in Morris et al. (2016), particularly at small temporal offsets, is driven by these induced correlations.

We used a neuro‐computational model to understand these previous observations better. Our model combines the retinotopic visual input with the proprioceptive eye position signal and CD signals in gain field maps. It is built on the assumption that eye position information is integrated over time, even though it may originate from different sources, each carrying incomplete eye position information, interacting in the gain fields of the dorsal visual system. Two interacting gain field maps LIP_PC_ and LIP_CD_ enable us to approximate the experiment of Morris et al. (2016) using our simulated LIP recordings as input data for a linear decoder. Our model's architecture allows us to address a potential conflict between Morris et al. (2012) and B. Y. Xu et al. (2012), as the neurons in the LIP_PC_ population align with B. Y. Xu et al.'s (2012) categorization of consistent LIP gain field neurons. This enables us to model the slow (delayed) PC update on LIP_PC_ after B. Y. Xu et al.'s (2012) observations of consistent PC‐driven gain‐field neurons. Further, our results are in accordance with predictive information observed in the parietal cortex by Morris et al. (2012, 2016). Through the lens of our model, the conflict between B. Y. Xu et al. (2012) and Morris et al. (2012) can be resolved by the comparably late contribution of proprioception signals in perisaccadic perception. However, both signals become temporally integrated into gain‐field neurons that implicitly encode stimulus location in different reference coordinate systems used for stimulus localization.

We should stress that, like Morris et al. (2016), the mere ability to decode for accurate eye position does not necessarily suggest that the brain uses a decoding‐based approach to facilitate perisaccadic perception. Like Morris et al. (2016), we use the decoder as a tool to explore the accessible information in the network under specific conditions. This does not mean that linear decoding is a practical or biologically plausible approach to perisaccadic visual perception. This is mainly because the decoder requires saccade‐dependent weights. A new set of weights would need to be trained to accurately decode a different saccade. Thus, the brain would need a large set of different decoders and the correct one being selected by the eye movement plan. Further, the decoder is trained in a supervised fashion using a correct eye position signal. One would need to speculate where such a signal would be available in the brain. Finally, accurate eye position decoding, if used for localization of briefly flashed stimuli, would require new answers to localization errors in those experiments. The fact that accurate decoding for eye position is possible does not immediately lead to novel predictions. Based on our model and its limitations, we propose two testable predictions.

First, the CD signal, which in our model carries the core of presaccadic and intrasaccadic information, plays a crucial role in predictive remapping and supports transsaccadic visual stability, while contributing to presaccadic mislocalizations in the direction of the saccade for flashed stimuli in total darkness (Ziesche and Hamker 2011). If the CD signal were suppressed, our model predicts the absence of most presaccadic mislocalizations in saccade direction and the emergence of pronounced intra‐ and postsaccadic mislocalizations in the opposite direction. Second, while Morris et al. (2016) successfully decoded past eye position up to 200 ms after the saccade, our model failed to produce a perfect decoding for past eye position. To achieve this, a decoder would require neurons in LIP_PC_ that update to the new eye position at consistent yet staggered delays, effectively encoding elapsed time through these fixed lags. Given that both models use similarly simple linear decoders, we propose that the key difference lies in the dataset: Morris et al. (2016) used real neural recordings. Morris et al. (2016) note that weight differences across temporal offsets were relatively smooth, suggesting that individual neurons typically do not sharply shift their firing at specific delays, as otherwise their decoder should have assigned strong offset‐dependent weights. We therefore predict that proprioceptive eye position signals in the dorsal visual stream update in a stable but distributed fashion: temporally regular at both the population and single‐neuron level, but contributing only partially to each neuron's overall activity.

Both Morris et al. (2016) and our results invite further questions into the nature and origin of perisaccadic mislocalization. A classical interpretation of a subset of those errors has been that the brain has no access to the exact eye position during saccade but relies on a sluggish eye position signal for localization of briefly flashed stimuli (Dassonville et al. 1992). However, our model and data from Morris et al. (2016) suggest that the dorsal visual system contains the required information to compute a non‐sluggish eye position signal. Available data suggests that a mixed representation of the eye and stimulus position appears already at the level of the primary visual cortex (Morris and Krekelberg 2019; Parker et al. 2022). Models using mixed representations replicated a large repertoire of data from perisaccadic perception experiments (Ziesche and Hamker 2011, 2014; Bergelt and Hamker 2016; Ziesche et al. 2017; Bergelt and Hamker 2019), suggesting that an explicit eye position signal is neither computationally required nor desirable.

Our framework utilizes the CD signal to predict the new stimulus position already prior to saccade (see, e.g., Figures 10 and 11). Experiments requiring subjects to localize briefly flashed stimuli (Georg et al. 2008; Honda 1993) probe the time window of the temporary coexistence of predictive and presaccadic signals, as seen in Figure 11A, resulting, according to our model, in systematic errors due to the presence of a pre‐ and post‐saccadic reference frame.

In conclusion, our study supports the view that the brain uses mixed representations of stimulus and different eye position signals. They are consistent with recent observations of studies testing for eye position coding in the parietal cortex (B. Y. Xu et al. 2012; Morris et al. 2012, 2016) and allow for replicating phenomena of perisaccadic perception as investigated in previous studies (Ziesche and Hamker 2011, 2014; Bergelt and Hamker 2016; Ziesche et al. 2017; Bergelt and Hamker 2019). Future experimental and computational studies are required to provide further insight into how such mixed representations enable perceptual stability while also giving rise to typical patterns of mislocalization observed in laboratory experiments.

## Author Contributions

**Nikolai Stocks:** formal analysis, investigation, methodology, software, validation, visualization, writing – original draft. **Fred H. Hamker:** conceptualization, formal analysis, funding acquisition, investigation, project administration, supervision, validation, writing – original draft, writing – review and editing.

## Conflicts of Interest

The authors declare no conflicts of interest.

## Peer Review

The peer review history for this article is available at https://www.webofscience.com/api/gateway/wos/peer‐review/10.1111/ejn.70207.

## Supporting information


**Table S1.** List of all projections, their effect, connection pattern, and parameters.
**Figure S1.** Decoder performance and weights for the current eye position, trained on a mixed LIP population.

## Data Availability

All original code has been uploaded to Github and is publicly available at https://github.com/hamkerlab/Stocks2025_Eye_Position_Decoder.

## References

[ejn70207-bib-0001] Bays, P. M. , and M. Husain . 2007. “Spatial Remapping of the Visual World Across Saccades.” Neuroreport 18, no. 12: 1207–1213. 17632269 10.1097/WNR.0b013e328244e6c3 PMC2531238

[ejn70207-bib-0002] Bergelt, J. , and F. H. Hamker . 2016. “Suppression of Displacement Detection in the Presence and Absence of Eye Movements: A Neuro‐Computational Perspective.” Biological Cybernetics 110, no. 1: 81–89. 26733211 10.1007/s00422-015-0677-z

[ejn70207-bib-0003] Bergelt, J. , and F. H. Hamker . 2019. “Spatial Updating of Attention Across Eye Movements: A Neuro‐Computational Approach.” Journal of Vision 19, no. 7: 10–10. 10.1167/19.7.10 31323096

[ejn70207-bib-0004] Binda, P. , G. M. Cicchini , D. C. Burr , and M. C. Morrone . 2009. “Spatiotemporal Distortions of Visual Perception at the Time of Saccades.” Journal of Neuroscience 29, no. 42: 13147–13157. 19846702 10.1523/JNEUROSCI.3723-09.2009 PMC6665185

[ejn70207-bib-0005] Binda, P. , and M. C. Morrone . 2018. “Vision During Saccadic Eye Movements.” Annual Review of Vision Science 4: 193–213. 10.1146/annurev-vision-091517-034317 30222534

[ejn70207-bib-0006] Colby, C. L. , and J.‐R. Duhamel . 1996. “Spatial Representations for Action in Parietal Cortex.” Cognitive Brain Research 5, no. 1–2: 105–115. 9049076 10.1016/s0926-6410(96)00046-8

[ejn70207-bib-0007] Dassonville, P. , J. Schlag , and M. Schlag‐Rey . 1992. “Oculomotor Localization Relies on a Damped Representation of Saccadic Eye Displacement in Human and Nonhuman Primates.” Visual Neuroscience 9, no. 3–4: 261–269. 1390386 10.1017/s0952523800010671

[ejn70207-bib-0008] Duhamel, J.‐R. , C. L. Colby , and M. E. Goldberg . 1992. “The Updating of the Representation of Visual Space in Parietal Cortex by Intended Eye Movements.” Science 255, no. 5040: 90–92. 1553535 10.1126/science.1553535

[ejn70207-bib-0009] Georg, K. , F. H. Hamker , and M. Lappe . 2008. “Influence of Adaptation State and Stimulus Luminance on Peri‐Saccadic Localization.” Journal of Vision 8, no. 1: 15–15. 10.1167/8.1.15 18318618

[ejn70207-bib-0010] Hamker, F. H. , M. Zirnsak , A. Ziesche , and M. Lappe . 2011. “Computational Models of Spatial Updating in Peri‐Saccadic Perception.” Philosophical Transactions of the Royal Society, B: Biological Sciences 366, no. 1564: 554–571. 10.1098/rstb.2010.0229 PMC3030832 21242143

[ejn70207-bib-0011] Honda, H. 1993. “Saccade‐Contingent Displacement of the Apparent Position of Visual Stimuli Flashed on a Dimly Illuminated Structured Background.” Vision Research 33, no. 5–6: 709–716. 8351842 10.1016/0042-6989(93)90190-8

[ejn70207-bib-0012] Klier, E. M. , and D. E. Angelaki . 2008. “Spatial Updating and the Maintenance of Visual Constancy.” Neuroscience 156, no. 4: 801–818. 18786618 10.1016/j.neuroscience.2008.07.079 PMC2677727

[ejn70207-bib-0013] Melcher, D. , and C. L. Colby . 2008. “Trans‐Saccadic Perception.” Trends in Cognitive Sciences 12, no. 12: 466–473. 18951831 10.1016/j.tics.2008.09.003

[ejn70207-bib-0014] Morris, A. P. , F. Bremmer , and B. Krekelberg . 2016. “The Dorsal Visual System Predicts Future and Remembers Past Eye Position.” Frontiers in Systems Neuroscience 10: 9. 26941617 10.3389/fnsys.2016.00009 PMC4764714

[ejn70207-bib-0015] Morris, A. P. , and B. Krekelberg . 2019. “A Stable Visual World in Primate Primary Visual Cortex.” Current Biology 29, no. 9: 1471–1480. 31031112 10.1016/j.cub.2019.03.069 PMC6519108

[ejn70207-bib-0016] Morris, A. P. , M. Kubischik , K.‐P. Hoffmann , B. Krekelberg , and F. Bremmer . 2012. “Dynamics of Eye‐Position Signals in the Dorsal Visual System.” Current Biology 22, no. 3: 173–179. 22225775 10.1016/j.cub.2011.12.032 PMC3277641

[ejn70207-bib-0017] Ostendorf, F. , and R. J. Dolan . 2015. “Integration of Retinal and Extraretinal Information Across Eye Movements.” PLoS ONE 10, no. 1: e0116810. 25602956 10.1371/journal.pone.0116810 PMC4300226

[ejn70207-bib-0018] Parker, P. R. , E. T. Abe , E. S. Leonard , D. M. Martins , and C. M. Niell . 2022. “Joint Coding of Visual Input and Eye/Head Position in V1 of Freely Moving Mice.” Neuron 110, no. 23: 3897–3906. 36137549 10.1016/j.neuron.2022.08.029 PMC9742335

[ejn70207-bib-0019] Pola, J. 2004. “Models of the Mechanism Underlying Perceived Location of a Perisaccadic Flash.” Vision Research 44, no. 24: 2799–2813. 15342224 10.1016/j.visres.2004.06.008

[ejn70207-bib-0020] Pouget, A. , S. Deneve , and J.‐R. Duhamel . 2002. “A Computational Perspective on the Neural Basis of Multisensory Spatial Representations.” Nature Reviews Neuroscience 3, no. 9: 741–747. 12209122 10.1038/nrn914

[ejn70207-bib-0021] Schlag, J. , and M. Schlag‐Rey . 2002. “Through the Eye, Slowly; Delays and Localization Errors in the Visual System.” Nature Reviews Neuroscience 3, no. 3: 191–215. 11994751 10.1038/nrn750

[ejn70207-bib-0022] Sommer, M. A. , and R. H. Wurtz . 1998. “Frontal Eye Field Neurons Orthodromically Activated From the Superior Colliculus.” Journal of Neurophysiology 80, no. 6: 3331–3335. 9862927 10.1152/jn.1998.80.6.3331

[ejn70207-bib-0023] Sommer, M. A. , and R. H. Wurtz . 2004. “What the Brain Stem Tells the Frontal Cortex. i. Oculomotor Signals Sent From Superior Colliculus to Frontal Eye Field via Mediodorsal Thalamus.” Journal of Neurophysiology 91, no. 3: 1381–1402. 14573558 10.1152/jn.00738.2003

[ejn70207-bib-0024] Sommer, M. A. , and R. H. Wurtz . 2008a. “Brain Circuits for the Internal Monitoring of Movements.” Annual Review of Neuroscience 31: 317–338. 10.1146/annurev.neuro.31.060407.125627 PMC2813694 18558858

[ejn70207-bib-0025] Sommer, M. A. , and R. H. Wurtz . 2008b. “Visual Perception and Corollary Discharge.” Perception 37, no. 3: 408–418. 18491718 10.1068/p5873 PMC2807735

[ejn70207-bib-0026] Sperry, R. W. 1950. “Neural Basis of the Spontaneous Optokinetic Response Produced by Visual Inversion.” Journal of Comparative and Physiological Psychology 43, no. 6: 482–489. 14794830 10.1037/h0055479

[ejn70207-bib-0027] Van Wetter, S. M. , and A. J. Van Opstal . 2008. “Experimental Test of Visuomotor Updating Models That Explain Perisaccadic Mislocalization.” Journal of Vision 8, no. 14: 1–22. 19146309 10.1167/8.14.8

[ejn70207-bib-0028] Vitay, J. , H. Ü. Dinkelbach , and F. H. Hamker . 2015. “Annarchy: A Code Generation Approach to Neural Simulations on Parallel Hardware.” Frontiers in Neuroinformatics 9: 19. 26283957 10.3389/fninf.2015.00019 PMC4521356

[ejn70207-bib-0029] Von Holst, E. , and H. Mittelstaedt . 1950. “Das Reafferenzprinzip: Wechselwirkungen Zwischen Zentralnervensystem und Peripherie.” Naturwissenschaften 37, no. 20: 464–476.

[ejn70207-bib-0030] Wang, X. , M. Zhang , I. S. Cohen , and M. E. Goldberg . 2007. “The Proprioceptive Representation of Eye Position in Monkey Primary Somatosensory Cortex.” Nature Neuroscience 10, no. 5: 640–646. 17396123 10.1038/nn1878

[ejn70207-bib-0031] Xing, J. , and R. A. Andersen . 2000. “Models of the Posterior Parietal Cortex Which Perform Multimodal Integration and Represent Space in Several Coordinate Frames.” Journal of Cognitive Neuroscience 12, no. 4: 601–614. 10936913 10.1162/089892900562363

[ejn70207-bib-0032] Xu, B. Y. , C. Karachi , and M. E. Goldberg . 2012. “The Postsaccadic Unreliability of Gain Fields Precludes the Motor System From Using a Simple Gain‐Field Algorithm to Calculate Target Position in Space.” Neuron 76, no. 6: 1201. 23259954 10.1016/j.neuron.2012.10.034 PMC3673542

[ejn70207-bib-0033] Xu, Y. , X. Wang , C. Peck , and M. E. Goldberg . 2011. “The Time Course of the Tonic Oculomotor Proprioceptive Signal in Area 3a of Somatosensory Cortex.” Journal of Neurophysiology 106, no. 1: 71–77. 21346201 10.1152/jn.00668.2010 PMC3129727

[ejn70207-bib-0034] Ziesche, A. , J. Bergelt , H. Deubel , and F. H. Hamker . 2017. “Pre‐ and Post‐Saccadic Stimulus Timing in Saccadic Suppression of Displacement–A Computational Model.” Vision Research 138: 1–11. 28709922 10.1016/j.visres.2017.06.007

[ejn70207-bib-0035] Ziesche, A. , and F. H. Hamker . 2011. “A Computational Model for the Influence of Corollary Discharge and Proprioception on the Perisaccadic Mislocalization of Briefly Presented Stimuli in Complete Darkness.” Journal of Neuroscience 31, no. 48: 17392–17405. 22131401 10.1523/JNEUROSCI.3407-11.2011 PMC6623809

[ejn70207-bib-0036] Ziesche, A. , and F. H. Hamker . 2014. “Brain Circuits Underlying Visual Stability Across Eye Move‐Ments—Converging Evidence for a Neuro‐Computational Model of Area LIP.” Frontiers in Computational Neuroscience 8: 25. 24653691 10.3389/fncom.2014.00025 PMC3949326

