# Supplementary material for: Bridging Conflicting Views on Eye Position Signals: A Neurocomputational Approach to Perisaccadic Perception: Eye Position Information in Brain and Model
Source: Eur J Neurosci. 2025 Aug 4;62(3):e70207. doi: 10.1111/ejn.70207 (PMC12320919; doi:10.1111/ejn.70207)
Supplement: Supplementary file 1 — Table S1. List of all projections, their effect, connection pattern, and parameters. Figure S1. Decoder performance and weights for the current eye position, trained on a mixed LIP population. [file EJN-62-0-s001.pdf]

Bridging Conflicting Views on Eye-Position Signals: A Neurocomputational Approach to Perisaccadic Perception

Authors: Nikolai Stocks, and Fred H. Hamker

Supporting Information

Projections

We use a multiplicative gain-field (see early work by Pouget et al. (2002)) population for coordinate transformation. Our population and projection geometry enables the efficient extraction of an emergent signal contained in the ordered proportion of two interactive base signals. We construct two dimensional gain-field populations for visual and eye position signals. The vertical axis encodes the position of a stimulus in a 1D space via Xr_{eye} and the horizontal axis encodes the eye position, either the proprioceptive eye position via PC_{eye} or corollary discharge via CD_{eye}. This arrangement of gain-field and input leads to ordered pairs of the two base signals of the same proportion occupying the same diagonal in the gain-field. Thus, both gain field maps can interact by calculating the sum for each main diagonal of the gain field, which we explicitly compute by the Xh population.

All weights of all projections between populations in this model are calculated using a simple Gaussian based on the distance index between pre- and postsynaptic neurons, with $K^{\text{pre, post}}$ as weight scalar and $\sigma^{\text{pre, post}}$ determining the Gaussians width.

$$w^{\text{pre, post}} = K^{\text{pre, post}} \exp\left(-\frac{\text{distance}}{(\sigma^{\text{pre, post}})^2}\right) \tag{1}$$

2D populations connect to other populations using a symmetrical projection along their diagonal axis, which produces a one-dimensional signal. To calculate the parameter *distance* in Equation 1, we only need to consider one dimension of the post-synaptic population. The *distance* is then calculated, depending on the geometry of the presynaptic population, in one of two ways:

$$distance = \|x - i\|^2 \qquad \text{(1D)} \tag{2}$$

$$distance = \|x - l - m\|^2 \qquad \text{(2D)} \tag{3}$$

with i as the index of a given neuron in a 1D presynaptic population, (l, m) as the indices in a 2D population and x as the relevant index of the postsynaptic neuron. There are two exceptions to this rule in this model. The first is the connection from Xh_{head} to LIP_{CD}, since the 1D population Xh_{head} projects to both dimensions of LIP_{CD}. However, the *distance* is invariant to the direction in which it is measured, so Equation 3 still applies, with x now as presynaptic neuron and (l, m) as postsynaptic neuron. The second exception is the self-exciting *distance* based projection of LIP_{PC} to itself, which is the one case in which we need to calculate the *distance* in 2D space proper:

$$distance = (\|x - l\|^2 + \|y - m\|^2) \qquad \text{(2D} \rightarrow \text{2D)} \tag{4}$$

with (x, y) as postsynaptic neuron and (l, m) as the presynaptic neuron. Self-inhibition is modeled as all-to-all connection with a uniform weight, so that $w_{inh}^{\text{pre, post}} = K_{inh}^{\text{pre, post}}$. The cutoff value for $w^{\text{pre, post}}$ is 0.001, below which no connections are formed in any projection.

To achieve the gradual staggered update of the proprioceptive signal observed by Xu et al. (2012), we apply a random delay to each synapse connecting PC_{head} to other populations. The delay is determined by sampling from a normal distribution with $\mu = 200$, $\sigma = 100$, a minimum value of $120ms$, and a maximum value of 250. It is important to stress that this does not represent a synaptic delay, but mimics the update latency in PC_{head}.

Populations

The sensory input for Xr_{eye} and PC_{head} follows the same general structure for a simplified point-like stimulus and is modeled by a Gaussian activation profile:

$$Input_i^{\text{Pop}} = c_{pop} \exp \frac{-\|p_{stim} - c_i^{\text{Pop}}\|^2}{2(\sigma^{\text{Pop}})^2}. \tag{5}$$

Projection	Effect	Geometry	K	σ
$Xr_{eye} \rightarrow LIP_{PC}$	*excitatory*	$1D \rightarrow 2D$ horizontal	1	0.075
$Xr_{eye} \rightarrow LIP_{CD}$	*excitatory*	$1D \rightarrow 2D$ horizontal	6	0.075
$PC_{head} \rightarrow LIP_{PC}$	*excitatory*	$1D \rightarrow 2D$ vertical	3	1.0
$PC_{head} \rightarrow CD_{head}$	*excitatory*	$1D \rightarrow 2D$ vertical	15	0.5
$CD_{eye} \rightarrow CD_{head}$	*excitatory*	$1D \rightarrow 2D$ horizontal	1	1.0
$CD_{head} \rightarrow CD_{head}$	*inhibitory*	$all - to - all$	0.2	NA
$CD_{head} \rightarrow LIP_{CD}$	*excitatory*	$2D \rightarrow 2D$ vertical	2	1.0
$LIP_{PC} \rightarrow Xh_{head}$	*excitatory*	$2D \rightarrow 1D$	0.3	2.5
$LIP_{PC} \rightarrow LIP_{PC}$	*inhibitory*	$all - to - all$	0.4	NA
$LIP_{PC} \rightarrow LIP_{PC}$	*excitatory*	$2D \rightarrow 2D$	0.6	0.5
$LIP_{CD} \rightarrow Xh_{head}$	*excitatory*	$2D \rightarrow 1D$	0.1	2.5
$LIP_{CD} \rightarrow LIP_{CD}$	*inhibitory*	$all - to - all$	0.2	NA
$Xh_{head} \rightarrow LIP_{CD}$	*excitatory*	$1D \rightarrow 2D$ diagonal	0.5	10
$Xh_{head} \rightarrow Xh_{head}$	*excitatory*	$1D \rightarrow 1D$	0.2	0.5
$Xh_{head} \rightarrow Xh_{head}$	*inhibitory*	$all - to - all$	1.0	NA

Table 1: List of all projections, their effect, connection pattern, and parameters.

With $\|p_{stim} - c_i^{\mathrm{Pop}}\|$ as the distance between the stimulus position and the receptive field center. c_{pop} is a tuning variable expressing stimulus strength or contrast with $c_{\mathrm{Xr_{eye}}} = 1$ and $c_{\mathrm{PC_{head}}} = 0.6$. To increase the spatial resolution and distinction between pre- and postsaccadic activation patterns, we restrict visual space to 80 degrees and set $\sigma^{\mathrm{Xr_{eye}}}$ to 3.175 and $\sigma^{\mathrm{PC_{head}}}$ to 5.

The input to CD_{eye} neurons follows a Gaussian profile and changes in strength over time:

$$Input_i^{\mathrm{CD_{eye}}}(t) \quad = \quad S_{\mathrm{CD_{eye}}}(t)\, c_{\mathrm{CD_{eye}}} \exp \frac{-\|c_i^{\mathrm{CD_{eye}}} - c^{\mathrm{CD_{eye}}}\|^2}{2(\sigma^{\mathrm{CD_{eye}}})^2} \tag{6}$$

With $c^{\mathrm{CD_{eye}}}$ as the retinotopic saccade target, $c_i^{\mathrm{CD_{eye}}}$ as the position of a given cells receptive field center, $c_{\mathrm{CD}} = 1.5$ and a $\sigma^{\mathrm{CD_{eye}}}$ of 1.

The temporal profile of the transient CD signal is determined by $S_{\mathrm{CD_{eye}}}(t)$. It consists of two Gaussians with different sigmas for the rise ($S_{\mathrm{CD_{eye}}} = S_{\mathrm{CD_{eye},\,on}}$) and decay ($S_{\mathrm{CD_{eye}}} = S_{\mathrm{CD_{eye},\,off}}$):

$$S_{\mathrm{CD_{eye},\,on}}(t) = \exp\frac{-\|t_{\mathrm{CD}} - t\|^2}{2(\sigma_{\mathrm{rise}})^2} \qquad (\sigma_{\mathrm{rise}} = 30) \tag{7}$$

$$S_{\mathrm{CD_{eye},\,off}}(t) = \exp\frac{-\|t_{\mathrm{CD}} - t\|^2}{2(\sigma_{\mathrm{decay}})^2} \qquad (\sigma_{\mathrm{decay}} = 54) \tag{8}$$

with $t_{\mathrm{CD}} = 10ms$, relative to saccade onset. It represents both the time of the maximal CD_{eye} activity and also the beginning of the CD signals decay, described by $S_{\mathrm{CD_{eye},\,off}}$.

The activation function of a given Xr_{eye} neuron i is described by Equation 9:

$$\begin{gathered} \tau * \frac{dm_i}{dt} + m_i = input_i - m_i * K * \sum_{n=1}^{40} input_n \\ r_i = [(m_i - threshold)]^+ \end{gathered} \tag{9}$$

With K = 0.524 as a constant value mediating the self-inhibition, and a minimum activity threshold of 0.05. $\tau = 10ms$ for all neurons in the network. Both the PC_{head} and CD_{eye} populations use the same simple activation function:

$$\tau * \frac{dr_i^{\mathrm{Pop}}}{dt} + r_i^{\mathrm{Pop}} = Input_i^{\mathrm{Pop}} \qquad (min = 0; max = 1) \tag{10}$$

The firing rate of the CD_{head} population is calculated using Equation 11.

$$\tau\frac{dr}{dt} + r = \sum_{\mathrm{FF1}} w^{\mathrm{CD_{eye},CD_{head}}} \cdot r^{\mathrm{CD_{eye}}} \sum_{\mathrm{FF2}} w^{\mathrm{PC_{head},CD_{head}}} \cdot r^{\mathrm{PC_{head}}} - \sum_{\mathrm{inh}} w^{\mathrm{CD_{head},CD_{head}}} \cdot r \tag{11}$$

Equation 12 determines the firing rate of the LIP_{PC} population:

$$
\begin{aligned}
\tau \frac{dr}{dt} + r = \sum_{\text{FF1}} w^{\text{Xr}_{\text{eye}},\text{LIP}_{\text{PC}}} \cdot r^{\text{Xr}_{\text{eye}}} (\sum_{\text{FF2}} w^{\text{PC}_{\text{head}},\text{LIP}_{\text{PC}}} \cdot r^{\text{PC}_{\text{head}}} + C + [A - r_{max}]^{+}) \\
-(r + D) \cdot \sum_{\text{inh}} w^{\text{LIP}_{\text{PC}},\text{LIP}_{\text{PC}}} \cdot r + \sum_{\text{exc}} w^{\text{LIP}_{\text{PC}},\text{LIP}_{\text{PC}}} \cdot r \\
+Noise \qquad (min = 0; max = 2)
\end{aligned}
\tag{12}
$$

The activity of the LIP_{CD} population is calculated similarly to LIP_{PC}, without the self-excitation but with the added gain modulated feedback from Xh_{head}.

$$
\begin{aligned}
\tau \frac{dr}{dt} + r = \sum_{\text{FF1}} w^{\text{Xr}_{\text{eye}},\text{LIP}_{\text{CD}}} \cdot r^{\text{Xr}_{\text{eye}}} (\sum_{\text{FF2}} w^{\text{CD}_{\text{head}},\text{LIP}_{\text{CD}}} \cdot r^{\text{CD}_{\text{head}}} + C + [A - r_{max}]^{+}) \\
-(r + D) \cdot \sum_{\text{inh}} w^{\text{LIP}_{\text{CD}},\text{LIP}_{\text{CD}}} \cdot r \\
+\sum_{\text{FB}} w^{\text{Xh}_{\text{head}},\text{LIP}_{\text{CD}}} \cdot r^{\text{Xh}_{\text{head}}} \sum_{\text{FF2}} w^{\text{CD}_{\text{head}},\text{LIP}_{\text{CD}}} \cdot r^{\text{CD}_{\text{head}}} \\
+Noise \qquad (min = 0; max = 2)
\end{aligned}
\tag{13}
$$

Both Equation 12 and 13 use the same set of parameters: $A = 0.5$, $C = 0.3$ and $D = 0.1$. $Noise$ is randomly generated using a normal distribution with a minimum value of 0.005 and a maximum value of 0.02.

The population Xh_{head} is computed by a set of equations:

$$
input = \sum_{\text{FF1}} w^{\text{LIP}_{\text{PC}},\text{Xh}_{\text{head}}} \cdot r^{\text{LIP}_{\text{PC}}} \sum_{\text{FF2}} w^{\text{LIP}_{\text{CD}},\text{Xh}_{\text{head}}} \cdot r^{\text{LIP}_{\text{CD}}} \tag{14}
$$

$$
\tau_s \frac{ds}{dt} + s = input \qquad (\tau_s = 10000) \tag{15}
$$

$$
suppression = 1 - D_s s \qquad (min = 0; max = 1) \tag{16}
$$

$$
\tau \frac{dr}{dt} + r = input \cdot suppression + \sum_{\text{exc}} w^{\text{Xh}_{\text{head}},\text{Xh}_{\text{head}}} \cdot r - (r - D) \sum_{\text{inh}} w^{\text{Xh}_{\text{head}},\text{Xh}_{\text{head}}} \cdot r \tag{17}
$$

with $D_s = 2.2$ and $D = 0.6$. When updating the firing rates all neural projections use a delay of $1ms$, i.e. a single simulation time step.

Mixed Network

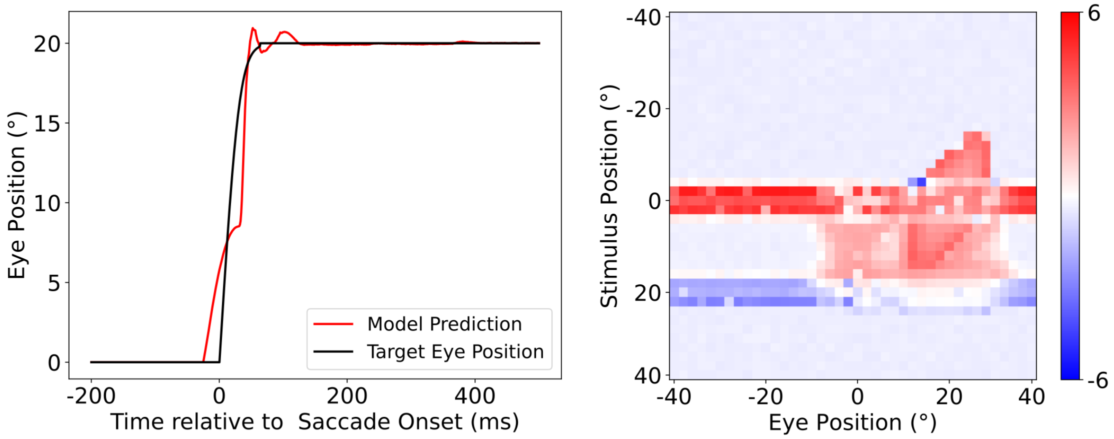

Supporting Figure 1: Decoder performance and weights for the current eye position, trained on a mixed LIP population.

To test to what extent our decoders' performance is dependent on the separation of LIP_{PC} and LIP_{CD} cells, we repeated the experiment with an altered LIP as input for the Decoder. From the two populations of 40x40 neurons, we constructed a single mixed LIP population with the following formula.

$$r_{ij}^{\mathrm{LIP}_{\mathrm{mixed}}} = A_{ij} \cdot r_{ij}^{\mathrm{LIP}_{\mathrm{PC}}} + (1 - A_{ij}) \cdot r_{ij}^{\mathrm{LIP}_{\mathrm{CD}}} \tag{18}$$

$$A_{ij} \sim \mathcal{U}(0, 1) \tag{19}$$

Figure 1 shows that the decoders performance decreases slightly, but well within what would be expected given the significantly noisier inputs and the smaller set of parameters, as the number of weights available to the decoder are only half compared to those of the original model.

References

Pouget, A., Deneve, S., & Duhamel, J.-R. (2002). A computational perspective on the neural basis of

multisensory spatial representations. *Nature Reviews Neuroscience*, *3*(9), 741–747.

Xu, B. Y., Karachi, C., & Goldberg, M. E. (2012). The postsaccadic unreliability of gain fields precludes the motor system from using a simple gain-field algorithm to calculate target position in space. *Neuron*, *76*(6), 1201.
